# Hospital and Institutional News

**Published:** 1912-10-12

**Authors:** 


### HOSPITAL AND INSTITUTIONAL NEWS.

### INSTITUTIONAL SOLIDARITY AND THE BIRMINGHAM CONFERENCE.

We conclude this week our full report of the proceedings of the British Hospitals Association at the Birmingham Conference by the publication of Mr. Edgar Kemp's paper on "The Training and Work of the Almoner" and the interesting discussion which ensued upon it. We would direct on this occasion, however, particular attention to the words of Mr. Howard Collins, whose speech as Honorary Local Secretary brought the proceedings of the Conference to a close. He held out in the first place a cordial, or, we would prefer to say, a statesmanlike invitation to all those who are eligible for membership to join the British Hospitals Association. This he enforced by a confession that goes to the root of the matter. "Some of us," he said, "are old enough to have had difficulties and messes. Those belonging to the Association believe that they can help others to avoid them." That, of course, is the meaning of institutional solidarity, and though very few individual hospital managers would ever desire to withhold information they possessed, this co-operative feeling requires a mode of expression. That, of course, is what the British Hospitals Association provides. It has a unique field, for, though other associations and societies exist, and rightly exist, in the country, there is none other which is all-embracing in its scope, and which, while attracting the best minds in the hospital world, allows, as the various discussions have shown, every institution, however small, to contribute its experience, and welcomes the opinion of every hospital officer whatever his degree.

### A CRECHE SUPERINTENDENT CENSURED.

The adjourned inquiry into the death of a child, who, with three others in as many days, died at a branch of the Walworth Crèche in Tooting, led to a verdict of "death from natural causes," the jury adding that the Superintendent, Mrs. Kinghorne, was deserving of severe censure. In the course of the evidence it appeared that Mrs. Kinghorne was not a member of the National Society of Day Nurseries, and one witness described her as "kind-hearted," "self-sacrificing," and "entirely given up to philanthropic work." How useless such qualities are for the care of infants, unless accompanied by expert training and organising power, the death-rate in this branch of the Walworth Crèche serves to show. The crèche itself was in charge of a "matron," who admitted that she was not a trained nurse, and of an "assistant" who was equally destitute of recognised training. The "matron" stated that the home consisted of two rooms and that there were no thermometers, while various statements were made to the effect that economy was greatly encouraged. Into further details it is unnecessary to go, and the broad facts remain that crèches of this kind, whose haphazard organisation is little short of criminal, and whose staff possess no technical training for their work, should not be allowed to exist. A lady inspector of the London County Council is said to have visited the home, but little seems to have been achieved by her visit. Pending much more drastic regulations for the opening of crèches generally, the public should be warned against all which are not affiliated to one or other of the recognised voluntary agencies whose certificates represent a genuine, if limited, standard of efficiency. It is satisfactory to learn, however, that the institution connected with the present inquest was to be closed on Saturday last, and it is much to be hoped that Mrs. Kinghorne will devote her philanthropic efforts to other channels where her failure to realise her responsibility may produce less disastrous results.

### A NEW HOSPITAL FOR BIRMINGHAM.

An announcement is made that Birmingham is to have a new special hospital for the treatment of epilepsy, paralysis, and nervous disease. The promoters argue that Birmingham should not be allowed to fall behind London, which possesses three special nerve hospitals, and that many of the patients come from the provinces. It is understood that a site has been secured in Bath Row, which is capable of adaptation, and that a public appeal will be issued very shortly. The names of those identified with the proposed institution are given as Mr. E. Gilbert Smith, the skin specialist, as chairman, Mr. H. J. Peart as honorary secretary, and Mr. Joseph Ansell as honorary treasurer.

### DOCTORS' FEES: SETTLEMENT ADUMBRATED.

The result of an interview between Mr. Lloyd George and those members of the medical profession, who, having been appointed by the Insurance Commissioners members of the Joint Advisory Committee, refused to resign at the request of the British Medical Association, is that he hopes to make known the decision of the Cabinet as to medical remuneration under the Insurance Act during next week. The Chancellor, whose visitors were on this occasion headed by Sir Clifford Allbutt, received on Friday last week those members of the Advisory Committees who represent insured persons. An official statement quoted the view that, in the event of increased provision being made, the Imperial Exchequer must be responsible for it, since other benefits would suffer if the increased provision were accumulated out of their funds. The alternative methods of providing medical benefit suggested in case agreement between the Government and the profession were found impossible, were either the handing over of the money payable for medical benefit, including any additional grant which the Government might decide to make, to the approved societies, to whom the task of providing medical treatment and drugs should be completely entrusted; or that the Government should undertake the organisation of a National Medical Service, which some speakers urged should provide for the requirements not only of the insured but also of their dependants. The almost unanimous feeling of the meeting was in favour of the organisation of a National Medical Service as the preferable alternative in the event of failure to arrive at an agreement. After the Government have come to their decision a further meeting of the Advisory Committee is expected.

### PHARMACISTS AND THE INSURANCE ACT.

The regulations respecting medical benefit under the National Insurance Act seem likely to form the subject of consideration by local pharmaceutical associations during the greater part of the winter session. So much depends, however, on the way in which the regulations are interpreted that until the Act comes into operation pharmacists will find it difficult to decide upon a plan of action. So far as the dispensing of medicines and the supply of drugs are concerned the regulations follow the Act closely, but the important question of prices has to be settled by the local Committees, some of which may show a tendency to effect too great an economy on their part of the administration of the Act. So long as the chemists hold together there is not likely to be much friction, provided, of course, the terms are strictly moderate; but there is the danger that for the sake of advertisement, or for some more obscure reason, pharmacists here and there may be disposed to offer to do the work at ridiculously low rates of remuneration. It is to be hoped, however, that any such demonstration of disloyalty will meet with no encouragement from the Committees, for it is obvious that if the pharmaceutical service is to be efficient it must be reasonably remunerated.

### THE PROPRIETARY MEDICINE INQUIRY.

The Select Committee which is inquiring into the sale of proprietary remedies will, it is expected, resume its sittings on October 17. A number of medical witnesses have yet to be heard, as well as witnesses representing patent medicine interests. It is understood that the Committee will be asked to hear evidence relating to infants' and invalids' foods, but unless the Government extends the scope of the inquiry the Committee will not have power to consider such evidence. In view of the fact that many of the prepared foods on the market are sold under false colours and at fictitious prices, it is unfortunate that the terms of reference to the Committee do not allow of an inquiry into this matter. Perhaps it is not too much to hope that the Government will widen the terms of reference.

### THE HEALTHINESS OF PANAMA.

The Master of Downing has issued an eloquent plea for emphasising the lesson which the present healthiness of Panama, so lately one of the most disease-ridden spots in the world, teaches to the public in England. He points out that the purging of Panama from all those causes which made it practically unfit for European habitation was accomplished in a short time by the concentrated endeavour of the American people, to whose benevolent despotism the present healthy conditions of the place are due. He reminds us again that this success is the result of no miraculous discovery, but merely of the concentrated application of methods which were the common knowledge of science, and the general scope of which at least was not unknown to laymen. Now, apart from the fact that it is refreshing to have other than political lessons drawn from the opening up of Panama, we may be grateful for the splendid illustration which its sanification gives of a very simple and very great truth. The Professor naturally laments that these methods cannot be imported into our public health work at home, and finds in the distraction caused by party politics a reason for the slow progress of sanitary reform. We fear that this reason is not a wholly sufficient explanation. The microbes and disease-carriers of Panama are represented in England by vested interests, which in a complicated society of enormous population like ours are so complexly intertwined that even reformers sometimes awake with a shock to find that their personal solvency is in some way bound up with the abuse which they wish to uproot. We have to deal over here with foes more elusive even than the microbes of malaria.

### THE NEW WING OF THE MILLER HOSPITAL.

In a month's time the new wing of the Miller General Hospital in Greenwich Road will be opened by the Duchess of Argyll, who is indefatigable in her support of charitable effort. This extension will give accommodation for fifty-one more patients. Besides this the new wing contains a theatre unit, which is placed on the top floor, and a new $x$-ray department. In addition, various modifications and improvements have been carried out in the kitchen department. The cost of the new wing and its equipment amounts to £23,000, of which some £16,000 have been subscribed, and the Trustees of the Zunz Bequest have promised £3,000 on condition that the remaining sum be raised. We shall hope to give at a later date further details of this extension, the accommodation of which is divided between three wards, each containing seventeen beds.

### MEDICAL OFFICERS AND THE INSURANCE COMMITTEES.

A rather curious difficulty has just been overcome at Burnley. Dr. Holt, Medical Officer to Burnley, was appointed under the Insurance Act consultative officer to the local Insurance Committee, and this appointment was permitted by the Town Council, whose whole-time officer Dr. Holt is, on condition that the salary of £100 attaching to the post should be paid to the Corporation. The Council's decision led Dr. Holt to resign, whereupon the Insurance Committee begged the Corporation to reconsider the matter. The health committee, before whom it came, recommended the Council to withdraw the condition which it had imposed on Dr. Holt's acceptance of the position, and last week the Council met to decide the matter. The Mayor, Alderman E. Keighley, pointed out that the medical officer was the most suitable person for the position, and said he hoped that the Council would adopt the recommendation of the health committee. Mr. Irving, on the other hand, said that Dr. Holt was a whole-time official, and that if he were allowed to take on and be paid for other work more responsibility would be thrown upon his assistants, who would soon ask for, and could not logically be refused, an advance in salary. Eventually it was decided to adopt the minute of the health committee. Practice appears to vary in different centres. Some medical officers undertake the duties without extra remuneration, and at others some one other than the medical officer of health is appointed to the post. Many, however, will sympathise with Dr. Holt in refusing to take on new duties without a suitable fee.

### PRIVATE CHARITY FOR PUBLIC BODIES.

It is sometimes said that no gifts are ever given to public bodies, and that authorities with public money behind them extinguish voluntary charity altogether. It is always instanced as a case in point that no estates are left for the benefit of the Imperial Exchequer, though conscience money is often known to find its way there. That this, however, is an exaggerated view is shown by the fact that the Mayor of Deptford, Mr. E. Mumford Preston, has offered £500 to the Borough Council for the provision of an institution for the use of the borough. This offer has been promptly seized on by the public health committee, which has recommended that the freehold of certain premises should be acquired and the balance of the money be devoted to adapting the premises for a tuberculosis dispensary. Anti-tuberculosis methods are now vieing with cancer research for the highest place in the public regard, with the resulting danger of an overlapping of effort. Doubtless the Deptford Borough Council has this well in mind, and has not decided on a tuberculosis dispensary without regard to other agencies in the locality. Mr. Preston's gift, however, is an interesting example of private charity given to the public authorities, who, all things considered, have perhaps the best means of applying it to the greatest advantage.

### DELIRIUM TREMENS AND INFIRMARIES.

The problem of dealing with cases of persons suffering from delirium tremens admitted to infirmaries has been dealt with by the Lambeth Board of Guardians. At one time the Lambeth Guardians took action in the Police Court, until the Stipendiary Magistrate's attention was drawn to the state of the law, and he had reluctantly to confess that he had no power to enforce payment of maintenance against patients suffering from delirium tremens. In order that the Board might be recouped in respect of the many "drink" cases admitted to their infirmary, and in the hope that it may prove a deterrent, the Guardians have framed a regulation enforcing on the person treated for delirium tremens or alcoholism the serving of a demand for the payment of the full cost of maintenance in the institution, and stipulate that he shall attend the next meeting of the full Board. In addition, they have laid down that persons admitted with sufficient money in their possession shall have the full cost of their maintenance deducted and the balance retained until personal application is made to the Board for it. In those cases where the patient fails to pay the Guardians' account the infirmary committee has been empowered to take proceedings in the County Court to recover the amounts. It is hoped by these means that the number of people seeking relief in the infirmary from over-indulgence in alcohol will be reduced.

### A HOSPITAL CRISIS AT STOKE-ON-TRENT.

The financial condition of the North Staffordshire Infirmary at Stoke-on-Trent is felt to be so serious that the general committee has issued a report which was considered at a special meeting on Wednesday last week. Two years have produced a deficit on the income of £6,600. The committee, therefore, recommends that securities for £10,000 should be deposited at the bank, with a view to their sale if the overdraft be not paid off by public subscription. A campaign is urgently needed for increasing the annual income by £4,000 a year, no light matter in these days, but one which the energies exhibited by the managers of King Edward VII. Hospital, Cardiff, has proved once more to be possible to enthusiasm and hard work. Mr. A. H. Heath, the president of the infirmary, is determined that steps shall be taken to resuscitate the financial condition into which the institution has been drifting of late, and it seems clear that an appeal to the public could no longer be delayed.

### LONDON'S WASTE OF AVAILABLE BEDS.

Professor W. R. Smith, vice-chairman of the Metropolitan Asylums Board, criticised in a vigorous speech last Saturday the clause in the Insurance Act which imposes a disability on the whole of the public health authorities, and prevents them from providing treatment for tuberculous patients in London. He pointed out that the Board had already expressed its willingness and capacity to place 800 to 1,000 beds at the disposal of the authorities, and now that tuberculosis had been made a notifiable disease it should be dealt with by the body now in charge of the other notifiable infectious diseases. By a sub-section of Section 16 the Board, however, was ruled out. Large numbers of patients were waiting to be treated, and by this clause, which ruled out "Poor-Law authorities," the Board, which though it had a Poor-Law origin, now occupied the position of a joint Hospital Board by providing infectious hospital accommodation for the twenty-nine local authorities in London, was prevented from using its hospital beds. Why, Professor Smith concluded, should the Metropolitan Asylums Board be stigmatised in this fashion, and why should London be put to the risk of incurring enormous expense through the operation of this clause, which excluded an already equipped organisation? A direct claim of this kind is a refreshing relief to the allegations of delay in providing sanatorium benefit and the tardy official denials which have occupied the newspapers of late. Certainly, if Professor Smith carries his point, other local authorities will be encouraged to see how they can adapt his hint to their own institutions.

### DUAL CONTROL AND HOSPITAL MANAGEMENT.

The recent re-election of Mr. H. R. Pink and the Rev. W. C. Hawksley as respectively chairman and vice-chairman of the governing body of the Royal Portsmouth Hospital was the occasion of an important proposal for the administrative reform of the institution. Mr. Pink said that as soon as Mr. Hawksley had passed the chair he hoped that steps would be taken to amalgamate the board of governors with the committee of management. He pointed out that the new reorganisation scheme had now to consider no longer the services of several elderly gentlemen which would have been lost had it been completed previously, since time had now removed them, and with this consideration no longer operative he hoped that the scheme would be quickly carried out. Mr. Hawksley also spoke in its favour, and said that he trusted that the governors would not wait until he had passed the chair. The present system of dual control has little to recommend it, and the present would seem a peculiarly happy time to abolish it, now that the discussions of the British Hospitals Association on the allocation of responsibility have been keeping the matter so much to the fore. The Royal Portsmouth Hospital must have learnt to appreciate for how much cost to institutional welfare defective administration is responsible, and the proposed change is therefore particularly welcome.

### WOMEN RESIDENT DOCTORS IN POOR-LAW INFIRMARIES.

Dr. Annie C. Greenop has been appointed by the Blackburn Board of Guardians resident assistant medical officer at the workhouse. The fact is worth putting on record, because it is another of the recent cases we have noted which have led to controversy. In August, we may remind our readers, the Dewsbury Board of Guardians proposed to appoint a woman resident doctor at their workhouse infirmary, but were discouraged by the Local Government Board on the alleged ground that no other institution as large as that at Dewsbury had been placed under the control of a woman doctor. The Board also alluded to cases with which a woman could not properly deal. The suggestion underlying this statement, however, was challenged in an article on "Women Resident Doctors in Poor-Law Infirmaries," which we published on September 7, by Dr. Catherine Kirk, who is the sole resident medical officer at the Poor-Law Hospital, Halifax, an institution with 400 beds. She pointed out that in her experience she had found no case with which a woman could not deal, and gave her reasons. These we venture to commend to Mr. Gerrard and to Mr. Michael Brothers, who, at the meeting of the Blackburn Guardians alluded to above, put forward objections of a similar character. Mr. Brothers alluded to the "rough kind of men who came into the workhouse suffering from all sorts of diseases," and Mr. Gerrard was in a minority of one in opposing the appointment. The number of women resident assistant medical officers is tending to grow, and it is to be desired that those who share the doubts of the two Guardians just mentioned should test them by referring to the practical experience of the work which is contained in our recent article.

### A LOSS TO "THE PRUDENTIAL."

The Prudential Assurance Company has been so much in the mouths of men during the past month or two that the death of their chief medical officer, Dr. Light, has an interest not merely medical. Dr. Light graduated at Cambridge in 1883, having studied at St. Thomas's Hospital and at Leeds. His appointments included resident posts at the Brompton Hospital for Consumption and at the Leeds General Infirmary. He was, of course, a member of the Life Assurance Medical Officers' Association, and contributed on several occasions to the medical newspapers. His death is stated to have been due to septic poisoning.

### THE WOMAN DOCTOR IN PUBLIC AFFAIRS.

In the interests of history we record that the husband of Dr. Elizabeth Wilks has been released from Brixton Prison, to which, it will be remembered, he was condemned for the non-payment of his wife's income-tax. The woman doctor is thus victorious over the Government; the release being a tacit admission of the injustice of holding a man legally responsible for the payment of taxes on an income beyond his control—an income, indeed, which the law itself has placed deliberately out of his reach by the Married Women's Property Act. We can claim this as a double triumph: a vindication of the profession's increasing activity in public affairs in general, and a successful example of the ability of the woman doctor to conduct affairs of importance to a successful issue in the face of official opposition.

### THE REWARD OF MEDICINE.

Now that rumours are current alleging the grasping nature of the medical profession, coupled with the rider that its members are already wealthy or at least highly paid men, it is worth while to devote more than cursory attention to the three latest doctors' estates which have been published. These, which chance alone has associated together, are a criterion not to be despised of the amount of money left by medical men generally. Taking the names of the deceased practitioners in alphabetical order we find a Guernsey surgeon, Mr. J. H. Larmouth, whose estate, purely personal apparently, amounted to £3,221. Then there is Dr. L. W. Richards, who may represent the monetary reward of foreign service, as he was formerly special plague medical officer to Bombay; he left net personalty of £1,189. Lastly, there is an F.R.C.S. of Derby, Mr. H. D. Shepherd, president of the Royal Medical Society of Edinburgh, whose net personalty is given as £2,812. We do not wish to exaggerate the importance of these three estates which chance has published simultaneously, but they represent fairly varied experience of typical classes of practitioners, and we question whether, say, the lawyers could often parallel estates so small in the various branches of their profession.

### THE "TROPICAL DISEASES BULLETIN."

With the fortieth number of the *Sleeping Sickness Bulletin* this periodical as such comes to an end. Edited ever since its commencement by Dr. A. G. Bagshawe, of the Uganda Medical Service, the *Bulletin* has prospered amazingly from the first, and has thriven to such an extent that it can no longer be carried on in the unusual conditions which have hitherto fostered its growth. The *Sleeping Sickness Bulletin* has been an official publication, supervised by a managing committee of Civil Service and other experts and supplied gratuitously to those who required it. It is now to be supplanted by a new periodical named the *Tropical Diseases Bulletin*, to be issued twice a month at a subscription of a guinea a year through Messrs. Baillière, Tindall and Cox. Single copies will be sold at one shilling and sixpence. Fortunately, the direction of the new *Bulletin* will remain in Dr. Bagshawe's hands; and he will be assisted by a departmental staff. The editor will be supported by Dr. G. C. Lowe, his assistant director at the Tropical Diseases Bureau. Each number of the new paper will contain about fifty pages of classified summaries of the current literature of tropical diseases. The tropical diseases of animals will be treated in a separate publication, the *Tropical Veterinary Bulletin*, a quarterly journal in charge of Mr. A. Leslie Sheather, B.Sc., M.R.C.V.S., published through the same agents as the *Tropical Diseases Bulletin*.

### DISTRICT PARASITES AND HOSPITAL WORK.

The Round-table Conference convened by the Mayor of Portsmouth for the purpose of bringing home to the outlying districts their responsibilities to the Royal Portsmouth Hospital is beginning to produce some result. The Havant Urban District Council has undertaken through its Chairman, Mr. Alfred Stent, to decide what can be done at its next meeting. The Alverstoke Board of Guardians has given notice that it will increase its subscription, and the Fareham Urban District Council has given notice of a resolution to ask the Local Government Board for permission to subscribe five guineas every year to the hospital. This, however, is, we hope, only a beginning. For, as an example of how much the latter district receives from the hospital and how little it does in return, we quote the statement of Mr. John Sandy that in the aggregate the cost of patients received by the hospital from Fareham is computed as £350, while the town's contribution to the hospital is £150 only. It is something more, then, than generosity that is required. For until the cost of patients from any district is compared with the amount of its subscription the inference in the lay mind always is that a generous contribution is the rule, and that further demands on generosity are asked for. The very reverse is the case. Generosity only steps in when just debts have been paid, and until they are paid the hospital can rest its claim upon its services. It is the hospital which has been the generous party. Far from being a perpetual beggar, as the voluntary hospital is sometimes called, it is the splendid disburser of favours. And every district which receives from the hospital far greater services than it subscribes for is a parasite on charity of a very dishonourable kind.

### CHISWICK MEDICAL OFFICER'S RETIREMENT.

Dr. Frederic C. Dodsworth, medical officer of health at Chiswick since 1878 and medical superintendent of Chiswick Isolation Hospital since its establishment about eight years ago, has sent in his resignation. The Chiswick District Council has, therefore, decided that his successor shall be required to devote his whole time to the duties required of him under the Public Health Acts at the isolation hospital and as medical inspector under the local education committee. The applicants for the post will include, we understand, two local medical men, Dr. Leaning and Dr. Littlejohn, who, some twelve months ago, with a view to gaining experience, were appointed assistant medical officers without salary.

### THE DECLINING NUMBER OF DEATHS UNDER ANÆSTHETICS.

Dr. Waldo, the City coroner, is tireless in his efforts to reduce the number of deaths under anæsthetics by urging the enforcement of wise regulations. As he reminded the jury at an inquest last week on a patient from Guy's Hospital, he had suggested that every death under an anæsthetic should be reported to the coroner, and that an open inquest before the coroner by jury should continue to follow necessarily in accordance with the law as at present constituted. The Home Office Departmental Committee, before which he gave evidence, left this, however, to the discretion of the individual coroner, by which means Dr. Waldo thinks a great protection is taken away from the public. The holding of coroners' inquests had given a publicity to deaths under anæsthetics which he thought undoubtedly had been productive of much good. Dr. Waldo also repeated his advocacy of the proper recording of statistics in all cases, and the sending of these by the coroner to the Registrar-General. At present such statistics were not only useless but misleading, and the Committee had not dealt with this important part of the question at all. In view of the remarks recently made in The Hospital on anæsthetics in American hospitals, it is interesting, if not surprising, that Mr. Thompson, of Guy's, who gave evidence at the inquest referred to, should emphasise the improvement for which greater care in selection and education of anæsthetists in our hospitals has been largely responsible. It is the technical education coupled with the publicity accorded by coroners' inquests which together have led to that gratifying decrease in the number of deaths under anæsthetics, on which Dr. Waldo also commented.

### CRIME AND MENTAL DISEASE.

At the opening of the October Sessions for the jurisdiction of the Central Criminal Court on Tuesday at the Old Bailey the Recorder, in his charge to the grand jury, had some interesting remarks to make on crime and mental disease. It was an alarming circumstance, he observed, that in a great number of cases coming before the Court of men charged with attempting to murder their wives the persons accused were clearly suffering from mental disease. He was constantly directing the attention of grand juries to crimes which had clearly been committed by men who ought to be in a mental hospital. This, however, the Recorder added, is a question for the doctors. Of the eighty-eight persons now awaiting trial, three are charged with murder, five with attempted murder, and two with manslaughter. There is no doubt that the psychology of crime is too little considered outside the text-books wholly devoted to the subject, and grand juries need the Recorder's warning, on which he was very wise to insist.

### A WARNING TO NURSING HOMES.

We have been asked to publish a warning to managers of nursing homes against a well-dressed man of gentlemanly appearance giving the name of Dr. Stawell and as address the Press Club. After offering Lady Curzon and Sir Alfred Fripp as references, he asks, our informant states, for a patient with acute kidney trouble, and an old friend of his, to be admitted as soon as possible, alleging that Sir Alfred Fripp has consented to operate upon him. Before leaving, the doctor makes a request for money, and inquiries have elicited the facts that he is unknown to the Press Club, and that Sir Alfred Fripp has only heard of him through the homes from which already he has attempted to borrow. It is stated that he is conversant with medical terms.

### CONSUMPTIVE CHILDREN IN BERLIN.

Considerable interest will be felt in the new scheme instituted by the Curatorium of the Berlin Heimstattenwesen under which special provision is made for the care of delicate children of the class formerly classed as "scrofulous," and now popularly styled "consumptive." We need scarcely point out to our readers that true phthisis pulmonalis is a very rare disease in children, and that what is commonly classed as consumption among them is the bovine or glandular-articular form of tuberculosis, a disease which readily yields to suitable treatment. The Curatorium has decided to build a communal children's home where these "delicate" children may be boarded, lodged, and educated until they are entirely cured. At Wilmersdorf the municipality has approved a similar scheme, and a site has already been purchased for the home at a cost of £20,000. The Aschersleben Curatorium has put aside £1,500 to serve as a special fund for such children, the object of which will be to enable some of the little patients to go to the baths and have free treatment at the Wilhelmsbad for a period of several months. In fact, all over Germany there is evident a tendency to deal more with the children, and to adopt preventive measures in fighting consumption. This seems to us to be the best method of attacking the disease. What we should like to see carried out in this country is the establishment of a number of seaside consumption camps, on the model of the prison and reformatory consumption camps in the United States. The cost of such a camp will be very low in comparison with that of a home or sanatorium.

### A HOSPITAL MEMORIAL TO A DYNASTY.

We have more than once stated that one of the foremost of Royal hospital workers of the present day is the Tsar of Russia. His Imperial Majesty's interest in hospitals and hospital work is very active, for he makes a point of yearly visiting every "Imperial" hospital in his Empire, and of having each institution specially inspected and reported on if he is unable to visit it himself. These reports are often the subject of detailed discussion with his entourage, and hospital workers in Russia know that if they can only bring a suggestion to the notice of the Little Father it is sure to have every consideration. This year, as is well known, the Tsar celebrates the 300th anniversary of his house as a ruling dynasty. With the Emperor's consent and approval M. Tagijew, the millionaire "oil King" of Baku, has given a large sum to build, equip, and endow a special hospital at Baku as a memorial to the 300-year reign of the Romanows. This will be the first hospital that stands as a memorial to a dynasty.

### THE WAR AND THE DRUG MARKET.

The unsettled political situation in the Balkans has not as yet had any very noticeable effect upon the Opium market, but there seems little doubt that the already high value of this drug will be increased not inconsiderably. Otto of roses is another product which would also advance in price in the event of war, notwithstanding that present quotations are higher than they have ever been. The Drug market has been more active during the week, but price changes of importance have not been numerous. Oil of aniseed is rather dearer. Oil of cloves has been advanced in consequence of the rise in value of cloves. Cod-liver oil is unaltered. Oil of eucalyptus still tends upwards in value. Oil of sandalwood is dearer. Balsam copaiba is rather lower. Balsam tolu continues very scarce. Higher prices are anticipated for juniper berries. Cantharides are dear, as also are chamomiles. Buchu leaves have advanced in price owing to the fact that the tax on collection at the Cape has been increased from 1s. 6d. to 2s. 6d. per pound. At the recent public sales in Mincing Lane, Cape aloes sold at dearer rates, coca leaves were cheaper, and ipecacuanha was dearer.

### TO OUR READERS.

Contributions are specially invited from any of our readers to these columns. They should deal with topical subjects and news. They must be authenticated for the information of the Editor only. The minimum payment if published is 5s. There is no hard-and-fast rule as to space, but notes of about twenty lines in length are preferred.

